# Experiential COVID-19 factors predicting resilience among Spanish adults

**DOI:** 10.1186/s40359-023-01131-4

**Published:** 2023-04-17

**Authors:** Mar Alcolea Álvarez, Natalia Solano Pinto

**Affiliations:** Department of Psychology, University of Castilla-La Mancha, Campus de la Fábrica de Armas. Av. de Carlos III, s/n, Toledo, 45071 Spain

**Keywords:** COVID-19, Resilience, Following mask recommendations, Vaccine obligation and perception of psychological health

## Abstract

**Background:**

The pandemic caused by COVID-19 has meant for spanish citizens a constant adaptation to health measures in order to try to stop transmission of the virus. During this adaptation process, different psychosocial aspects have caused consequences for people?s mental health to a greater or lesser extent. Makes sense of an emotional torrent who has gone through fear, anxiety, loneliness and anger. The interaction between perception and reality has given rise to situations where loneliness and social isolation have been imposed and lived with a load of emotional discomfort. In others, social isolation and measures to stop the pandemic have been accepted as a protection system and has been experienced since serenity and the feeling of self-protection fostering individual resilience. Studying the predictors of resilience is going to be key since it is the ideal antidote to stop the appearance of mental disorders associated with the pandemic (such as depression, anxiety, post-traumatic stress, social phobia, cleaning obsessions, and generalized anxiety disorder). The objective of this research is to analyze the relationship between resilience and experiential COVID-19 factors.

**Methods:**

Sample was comprised of Spanish adults (n = 1000; age 18-79 [mean =40.43],793 female, 201 male, and 2 non binary sex). These people participating in an online study focused on the impact of COVID-19 experiences. The research has been cross-sectional, descriptive and correlational design. The instrument created for this research was a specific online questionnaire, including the “Scale of resilience” (RS, Wagnild & Young, 1993, Spanish version, Sánchez-Teruel, et al., 2015). That questionnaire has been administered during the months of April 2022 to July 2022.

**Results:**

The results obtained show how people who have been able to face the pandemic in a responsive and adaptive way have high resilience. Specifically, those participants that accepting the use of masks, vaccinations and confinement obtained high resilience.

**Conclusions:**

Using public funding and allocating research to the development of programs to promote resilience, adaptative beliefs and prosocial behaviors becomes basic to live in a world in constant change.

**Supplementary Information:**

The online version contains supplementary material available at 10.1186/s40359-023-01131-4.

## Introduction

The COVID-19 pandemic provides an excellent opportunity to gain a greater understanding of relevant developmental processes that may contribute to keep mental health in adults in relation to their resilience and prosocial and responsive attitude.

All the efforts of this study go around this question: “Would it be possible to build strategies to evaluate and develop psychological variables that protect resilience in the face of adversity?”

The first time the term “resilience” was used in the science of psychology was thanks to the authors Werner and Smith [1]. As early as 1955, Werner evaluated nearly 700 newborns at the Hawaiian Islands. Subsequently, he conducted a longitudinal study including approximately 200 of those children, who came from disadvantaged socio-family backgrounds, and to whom a negative and precarious future was predicted. Almost thirty years later, Werner discovered that a moderate high percentage of those evaluated children had become adults with a normalized life, with structured families and high life satisfaction. Werner and Smith called these subjects “resilient”. Later, other authors, [2] also focused their attention on resilience as an aspect that exerted in people a high mental strength, with high autonomy, extraversion, sense of humor, creativity and values.

Decades of studies affirm the relationship between people with high resilience and adequate coping, and potentially traumatic situations; even with the development of even greater mental strengths after suffering events such as natural disasters or pandemics. For example, recents works [3, 4] explained how most people become stronger fighting the difficulties they face through psychological resilience. Resilience is a psychological construct that has been researched for many years, and there is a lot of literature on it. However, the relationship between resilience and the current pandemic is something much less studied that needs to be addressed in order to create mental health improvement programs in times of health crisis. It is essential to continue developing studies that offer explanations of how socio-demographic factors can affect the development of greater resilience and mental strengths, and focusing our efforts on creating prevention programs for risk situations and, above all, with the view of protecting and detecting populations at risk.

Something widely studied is the possible difference in resilient capacity according to gender. Is there greater resilience in men, women or both? According to the bibliography consulted by the researcher Luna et al., [5–7] and her team from the Chicago Medical School, there are no significant gender differences with respect to the resilience construct. They found more individual differences associated with personal life histories than the fact of being male or female.

Schmidt et al., [8] created, developed and validated the COVID-19 Impact Battery (CIB). In this battery they used two samples (the first one with 249 participants and the second one, 1000). This important study supposed to be comprehensive with validated measures in order to ensure systematic, consistent, and generalizable empirical work to measure the impact of COVID-19 on mental health and well-being. They developed a reliable and valid battery, which consisted of three scales assessing behaviors, worry, and disability related to COVID-19. Aspects such as cleanliness, avoidance or storage of sensors financial and health concerns, were part of evaluated behaviors. They were able to create a short version of this battery (CIB-S) so as to allow greater agility in data collection. In short, they were able to have two scales that collected the impact of the pandemic on the general mental health of the population.

Others studies [9] reported the development of a framework for resilience to disasters, such as a tornado or the COVID-19 pandemic. Conclusions were evident: the development of resilience directly affects the good coping with potentially traumatic situations; even after having personally suffered these adversities, individuals claimed to be more psychologically prepared for future natural catastrophes or crisis situations.

Cusack et al., [10] have investigated the resilience as a protective factor during the COVID-19 pandemic. They created a longitudinal investigation and they found resilience as a buffer against posttraumatic stress disorder (PTSD) and alcohol consumption. Overall, they could demonstrate a significant interaction between the COVID-19 worry impact domain and baseline resilience on later posttraumatic stress disorder symptoms.

Wearing a mask, respecting social distance or using vaccines have been behaviors that have protected us against the spread of COVID-19 virus. However, the psychological consequences of these measures in the short and long term are being studied and many remain to be analyzed. Olivera-La Rosa et al., [11] carried out an interesting study relating the fact of wearing a face mask and how people with sensitivity to disgust and social anxiety would perceive those faces as less trustworthy and more likely to be sick; and, therefore, tend to social distance. They confirmed their hypotheses.

With the same concern and focus., more researchers [12] studied on positive psychology and the development of strengths. They specifically studied the impact of the development of positive psychological capital in young people under 30 years of age that could improve their mental health. Psychological capital is made up of factors such as optimism, resilience, hope, and self-efficacy expectations. Their results pointed to the need of developing programs to enhance psychological capital and its direct impact on behavior and adaptive emotions of young people during the pandemic.

Biber et al., [13] were pioneers in studying how gratitude, optimism and factors related to resilience negatively correlated with the perception of anxiety during the first academic year of the COVID-19 pandemic. Their results strongly corroborated this hypothesis. In the same line of research more researchers [14] based their efforts to evaluate how a grateful behavior improves the coping and mental health during crisis or traumatic situations. Their outcomes showed that grateful people who experience gratitude frequently can buffer some negative psychological impact of the pandemic. Experiencing gratitude can sensitize us to perceive the world around us more intensely, something similar to what happens when we develop post-traumatic growth. This research, however, did not conclude that gratitude directly protected against adverse situations, but it did facilitate perceiving positive aspects of life, even in difficult situations, such as the pandemic.

Gratitude and resilience are strongly associated to constructs that can be predicted through specific behaviors, especially in times of adversity and difficulty.

Martinez-Martí, et al., [15] confirmed with their analysis that character strengths predict mental health and subjective well-being during difficult events. They conducted a longitudinal study with surveys completed by participants referring to different socio-demographic aspects and the 24 main personality strengths.

Krok et al., [16] showed how self-efficacy and meaning in life were associated with mental well-being and greater resilience. Seeing in his study how the pandemic is processed as a potentially traumatic and stressful event, at a cognitive and affective level in health workers.

Soon after that, relevant French authors at the University of Poitiers, as Goutaudier et al., [17] worked a 1-year longitudinal study with French people. Mainly, they researched about negative affective experiences during COVID-19 pandemic lockdown and posttraumatic growth. Their findings suggested that some people who had difficulty coping with the first lockdown (March/May2020) experienced subsequent growth one year later, and appeared to develop some form of mental strengths and post-traumatic growth.

It is very important to measure how COVID-19 pandemic affected more to old people that other range of age, and see how to improve their psychological factors for protection. Indeed, we can find references about “gero-pandemic”. How and why do some adults and communities adapt and thrive better than others? Differents studies [18] tried to answer that question because they found significantly higher morbidity and mortality risks during this COVID-19 crisis.

On the same way of research, KaapDeeder et al., [19] investigated associations between very varied resilience sources, like ego integrity, satisfaction need, perceived incomes, marital status, education level, medical conditions, and other relevant social-demographics aspects. Their results showed that some adults (e.g., those with a higher perceived income) were more resilient in coping with this crisis, and people with more hope and ego integrity could suffer less to across the pandemic.

Pretince, et al., [20] examined through a detailed study genetic aspects of Emotional Intelligence and the clear relationship between the traits of an emotionally intelligent person, as well as adaptive personality traits, and adequate coping with the pandemic, showing enlightening results of this hypothesis.

Another relevant aspect to review all the research with the evaluation of the psychological impact of the pandemic would be the detailed analysis of the development of prosocial behaviors, volunteering and attitude of helping others in adverse situations.

Wakefield, et al., [21] used data from the second and third waves of a three-wave survey to explore and evaluate psychological variables within volunteering and aid-giving. They saw a positive correlation between people who was engaged in prosocial and volunteer behaviors throughout their life history and in the first waves of the pandemic, and the probability of doing so later on other crisis or throughout this same pandemic that we were going through.

About the workers’ behavior, more researches, as Wu, et al., [22] found that some overqualified workers had greater difficulties in sacrificing, volunteering and performing low levels of extra-role, especially when the organization’s leaders had low self-sacrificial leadership during the COVID-19 pandemic. Or, in other words, high self-sacrificial leadership will activate employees sense of collectivism toward the organization. Further, this sense of collectivism will attenuate the negative association between perceived overqualification and felt obligation.

Bozdag & Ergün [23], despite the limitation of having a relatively small sample at the study (approximately 200 healthcare workers, mostly women), the study concluded with very significant results: workers with basic needs covered, such sleeping, life satisfaction and resilience, were able to provide a higher quality service at their work during the pandemic. These workers were able to better perform their duties, although despite following the recommendations to prevent COVID-19 spread.

Jokiü-Begiü et al., [24] wanted to study whether the psychiatry profession had different outcomes in properly and respectfully coping with the pandemic compared to other doctors. They were able to verify how younger workers, with less mental flexibility and less resilience, suffered greater stress during the pandemic and showed less resilience. They did not find that the sample of psychiatric professionals they evaluated showed a higher perception of stress than other physicians.

Analyzing how variables, such as using masks, vaccinations and confinement, increased adequate mental health, specifically predicting resilience, is the basis of all the reviewed researches.

Most of scientific authors about resilience´s factors [25] were focused on demonstrating the impact between high levels of resilience and the preservation of mental health in times of crisis. However, we observed that it was necessary to see if the acceptance of the measures implemented by the Spanish government could predict a high resilience to some extent. We have not been able to glimpse this specific aspect in the most recent studies found in relation to the pandemic.

In order to carry out the research, a rigorous study of the most current scientific literature related to resilience and the attitude observed during the pandemic was initiated.

This research aims to investigate how behaviors being the key to curbing the transmission of the virus predict a high resilience in people. Resilience, despite being a complex and difficult construct to analyze, has a positive and direct relationship to people’s mental health; this is why it is necessary to focus on its development. The main goal of the recent research already reviewed that has been reviewed focuses on assessing the psychological variables that favor good adaptation in crisis and catastrophes, such as the pandemic. In particular, the study has focused on seeing the predictability of resilience through attitudes towards government measures proposed to curb COVID-19, such as vaccination, use of face masks, or confinement.

This study marks a difference with respect to other investigations. The reason is that it focuses its emphasis on the relationship that exists between receptive attitudes towards the global measures of the World Health Organization and the ability to emerge strengthened from the pandemic.

Resilience means learning and overcoming situations that cause suffering, and therefore means preventing mental illness caused by the pandemic. The research shows that prosocial behaviors and civic attitudes linked to anticovid measures can predict resilience. This is therefore a basic aspect to see how people overcome the negative psychological effects of the pandemic thanks to these variables.

In addition, responsible attitudes, following the measures to stop the virus, become protective factors in the face of the catastrophe that the COVID-19 virus has brought on the world population.

The conclusion is basic, the more resilience a person can have, the less negative psychological impact they will have after suffering a crisis, catastrophe or emergency.

Therefore, analyzing the behaviors and attitudes that precede high resilience is the key to the success of this research.

It is highly important to invest in educational and preventive programs in order to promote resilience as to prepare population for an increasingly less predictable future. Do not doubt about the need of verifying the negative psychological impact of the pandemic on the Spanish population; however, we want to positively look into the future with the purpose of searching protective factors against any new emergency or catastrophe that we may suffer.

This study is based on a model where the dependent variable is resilience and the independent variables are: socio-demographic factors, safety, following mask recommendations, feeling strong, vaccine obligation and perception of psychological health.

Therefore the aim of the study of COVID-19 Experiential factors in the Spanish adult population, is to predict their levels of resilience, serving resilience as a balm against suffering, while said civic, decisive and collectivist behavior can help us to face possible negative circumstances that may arise.

It is also hypothesized that people who agreed about the mandatory nature of vaccination showed more resilience and finally. They also expressed that the pandemic made them psychologically stronger, scored higher in resilience.

## Methods

The research was carried out through cross-sectional, correlational and descriptive study. Adults were contacted and provided with information regarding the study. The online survey took approximately 15 min to be completed.

The study was a cross-sectional study of people of age who were invited to participate in a survey regarding COVID- 19 experiences and resilience. The inclusion criteria were: being over 18 years and having technological devices. 1300 people were invited to participate in the research through emailing and use of social networks. Only 1000 of them completed the survey.

The final sample size was 1000 participants (793 women, and 205 men). Mean age was 40.43, standard deviation 12.24, min and max 18–79 years (between 18 and 30 years, 208; 31–45, 442; 46–60, 305; more than 60 years, 45). 190 (19%) of participants lived in populations with less 5000 inhabitants; 445 (44.5%) in populations between 5000 and 50,000 inhabitants; and 365 (36.5%) in populations with more than 50,000 inhabitants. Regarding COVID-19, 511 (51.1%) of participants have suffered COVID-19 before the survey was done, 50.7% men y 50.2% women. From them, 115 (55.7%) were between 18 and 30 years; 236 (53.3%) between 31 and 45; 135 (44.2%) between 46 and 60, and 23 (51%) more than 60 years. Besides, 233 (45%) who had their residence in populations between 5000 and 50,000 inhabitants suffered COVID; 190 (37%) in populations with more than 50,000 inhabitants, and 88 (17.2%) got infected living in populations with less than 5000 inhabitants. Measures are described below.

### Procedure

Participants were informed about research objectives and the requirement of voluntary and anonymous participation. For this study, adult people were examined. Assessments for the study occurred during the following time periods: study was designed during summer, autumn and winter 2021, surveys were completed from April until July 2022.

### Measures

***Socio-demographic.*** Participants were asked about sex, age and occupation. Covariates. Participants were asked about having suffered from COVID 19, disability, health profession, being religious, mental health, and if family loss as well.

***Safety with masks.*** To measure safety with masks, an item was used (that it to say “*wearing the mask makes me feel safer about my health”).*

***Feeling strong.*** An item was created to assess feeling strong. (that it to say “*Thanks to what I have experienced due to the pandemic, I feel psychologically stronger than before”).*

***Vaccine obligation***. To measure the belief that vaccines should be mandatory, an item was used (that it to say “*I think vaccines should be mandatory”).* Each item, safety with masks, feeling strong, and vaccine obligation were rated using a 1–5 scale, wherein 1 and 5 meant *“totally disagree*” and “*totally agree,”* respectively.

***Following mask recommendations.*** An item was created to assess mask recommendations. (that it to say “*If I take my mask off in most places… I have used it when it was time to use it and now, I do not use it in most places because that is how it is established, in Spain, since April 20th, 2022”*).

***Perception of psychological health.*** To measure perception of psychological health, an item was created (that it to say *“I consider that my state of psychological health since pandemic is worse due to pandemic itself and the consequences it had for me*”). Each item, following mask recommendations and perception of psychological health, were rated using a the answers, yes or not.

***Resilience.*** It was measured with the Resilience Scale [25, 26]., which includes 14 items in the original and Spanish version questionnaires. Each item was rated using a 1–7 scale, wherein 1 and 7 meant *“totally disagree*” and “*totally agree,”* respectively. This scale showed excellent internal consistency in our study, with a Cronbach’s α of 0.90 in total sample, 0.91, and 0.90, in men and women respectively.

### Analytic approach

First of all, about analytic approach, descriptive statistics were conducted. Secondly, so as to determine the relationship between resilience (i.e., resilience score) and potential predictors (i.e., sex, age, safety with masks, feeling strong, vaccine obligation, following mask recommendations, perception of psychological health), were calculated by bivariate Pearson correlations. Finally, the influence of each potential predictor was estimated using multiple regression analysis. To avoid multicollinearity, given that some of the measures could be interrelated, only those variables which showed a significant correlation with participants’ resilience were included as predictors in the subsequent multiple regression analysis. Moreover, to ensure that there was no multicollinearity among these predictor variables, we used the variance inflation factor (VIF). VIF values between 1 and 10 are typically used to indicate the absence of multicollinearity of Cohen et al.; [27]. Data were analyzed using Statistical Package for Social Sciences, SPSS (Windows version 25).

## Results

Results could confirm that the studied COVID-19 experiential factors (socio-demographic, safety and following mask recommendations, feeling strong, vaccine obligation and perception of psychological health) predicted resilience among Spanish adults.

Descriptive statistics for all the studied variables are presented in Table 1 (categorical variables) and Table 2 (continuous variables).

### Women’s features

This sample showed a mean age of 39.9 years (ranging from 15 to 79). Most of women, 406 (51.2%), suffered COVID-19 and 188 (23.7%) lost a close relative due to COVID 19. However, 279 (35.2%) felt that their psychological health was worse due to the pandemic. Regarding use of masks, 291 (36.7%) women usually followed government recommendation about masks.

### Men’s features

Men showed a mean age of 42.4 years (ranging from 17 to 75). Most of men, 104 (50.7%), suffered COVID-19 and 49 (23.9%) lost a close relative due to COVID 19. However, 41 (20%) had a perception about state of psychological health is worse due to the pandemic. Regarding use of masks, 81 (39.5%) men usually followed government recommendation about masks.

**Table 1:** Demographic features and women and men’s characteristics (categorical variables)

		Number	Percentage
Women			
Occupation	Employed	431	54.3
	Housewife	178	22.5
	Student	101	12.7
	Student and employed	67	8.4
	Retired	16	2
Disability	Refers disability situation	27	3.4
Health professional	Being health professional	190	24
Religion	To be religious	219	27.6
Mental health	Refers to have mental disorders (i.e. depressions, anxiety, personality disorders)	217	27.4
Men			
Occupation	Employed	124	60.6
	Househusband	29	14.2
	Student	29	14.1
	Student and employed	9	4.4
	Retired	14	6.8
Disability	Refers disability situation	9	4.4
Health professional	Being health professional	16	7.8
Religion	To be religious	60	29.3
Mental health	Refers to have mental disorders (i.e. depressions, anxiety, personality disorders)	31	15.1

**Table 2:** Women and men’s descriptive statistics (continuous variables)

		Mean	SD	Min-max	Range
Women	Safety with masks	3.28	1.35	1–5	-
	Feeling strong	2.5	1.17	1–5	-
	Vaccine obligation	3.36	1.45	1–5	-
	Resilience	38.24	6	22–64	14–98
Men	Safety with masks	2.87	1.36	1–5	-
	Feeling strong	2.81	1.11	1–5	-
	Vaccine obligation	3.51	1.40	1–5	-
	Resilience	36.56	5.93	21–52	14–98

**Table 3:** Pearson correlations between resilience and the other variables of interest

	1	2	3	4	5	6
1.Resilience	-					
2.Age	− 0.097**	-				
3.Vaccine obligation	0.279**	0	-			
4.Feeling strong	0.142**	0.05	0.096**	-		
5.Safety with masks	0.277**	0.145*	0.297**	0.077*	-	
6. Sex	0.112**	− 0.08*	− 0.04	− 0.10**	0.12**	-

Note: * = p < .05; **=p < .01

Bivariate Pearson correlations with participant’s resilience scores are presented in Table 3. Higher scores of resilience are related to higher potential predictors (i.e., vaccine obligation, feeling strong and safety with masks and sex). However, higher scores of resilience are negative related to participant’s age. Additionally, no multicollinearity is evident among all other tested predictors, as evidenced by the VIF for the predictors, which ranged between 1.04 and 1.20, and tolerance values, ranged between 0.83 and 0.94.

For our main analysis, we conducted a multiple linear regression analysis with resilience as the target variable. The rest of the variables (i.e., aged, safety with masks, feeling strong, vaccine obligation, following mask recommendations, perception of psychological health) were included as predictors.

The results of the regression analysis are presented in Table 4, including standardized regression coefficients (βs) and the change in R^2^ for each predictor. The model accounted for 21% of the total variance in resilience (*F* = 39.122, *p* < .001). Vaccine obligation, perception of psychological health, feeling strong and safety with masks accounted for 7%, 12%, 16%, 19%, 20% and 21% respectively. Following mask recommendation was not significant (*ps* > 0.05).

**Table 4:** Multiple regression analysis to determine the influence of each predictor on resilience

	Resilience						
	B	SE	β	*P*	*R* ^*2*^	95% CIs	
						Lower bound	Upper bound
Vaccine obligation	0.874	0.124	0.210	0.000	0.077	0.632	1.117
Perception of psychological health	3.283	0.385	0.254	0.000	0.126	2.527	4.038
Feeling strong	1.015	0.154	0.197	0.000	0.162	0.714	1.317
Safety with masks	0.846	0.134	0.191	0.000	0.191	0.582	1.109
Age	− 0.063	0.014	− 0.127	0.000	0.207	− 0.090	− 0.035
Sex	1.150	0.432	0.077	0.000	0.212	0.303	1.998

As seen in the model, although relevant, it only explains 21% of the variability of resilience (dependent variable). Therefore, it is necessary to continue analyzing more variables, that have not been taken into account in this study in order to predict resilience, and which must be included for future studies. This model presents significant variables and the results indicate that all are predictive, and in the case of age with a negative relationship.

Regarding the weight of each of the variables in the model, the most relevant ones have been the perception of mental health, specifically feeling that psychological health is worse with the arrival of pandemic, followed by the variable of believing in the mandatory nature of vaccine, and finally feeling strong and safe with the use of mask. As socio-demographic factors, we saw a significant relationship between being young and a woman, and having a high resilience during pandemic. However, one must be cautious in the analysis of this last result, since the sample size of the sexes was not balanced. On the other hand, the “non-binary” group is not considered in this analysis due to the minimum sample size it presents (2 subjects).

## Discussion

It is essential to emphasize that this research based its hypothesis on how the independent variables (socio-demographic features, safety and following mask recommendations, feeling strong, vaccine obligation and perception of psychological health) predicted high levels of resilience. However, the results found, despite being the expected ones, the presence of different limitations. It is necessary to continue with the study, analyzing the variables predicting resilience beyond those studied so far, in order to have a wide range of explanatory factors and a high explanatory percentage. Safety with masks, feeling strong due to pandemic, the belief that vaccines should be mandatory, considering the state of psychological health to be worse due to pandemic and its consequences, are predictors of high resilience. In addition, being a woman and being younger also predict more resilience compared to being a man and being older. The variables “age” and “sex”, although they show significant conclusions, must be extrapolated with great caution. When carrying out an online questionnaire administered by massive emailing and social networks, a greater participation of women and adults around 30 and 50 years of age was evidenced. This can bias the analysis, so it is preferable to be more cautious and not draw hasty conclusions until progress is made in the data analysis.

Also, removing the mask following government recommendations or practice a religion (in case of women) is not related to resilience. What can be the hypotheses of these so statistically strange facts? First of all, we find the problem of sample validity, which has to do with the difficulty of having frames representative samples where each subject has an equal probability of being selected in the study it can happen that the stakeholder bias in obtaining certain results contributes to the massive participation of people oriented in the same beliefs or answers.

Despite the existing inequality between men and women due to the still sexism of the patriarchal society, the rate of participation in this research by women is impressive. The possible digital divide by gender has shown results contrary to what was expected. The hypotheses that we consider are related to the research theme, since being a questionnaire that contains items related to emotions, feelings, expression of beliefs and thoughts, it may be more attractive to women.

Also, the researchers themselves may believe that their social circle is made up mostly of women, which is why more women than men have had access to the administered questionnaire.

Regarding the fact of practicing a religion, the sample had a practically equal distribution of religious and non-religious people, without this being a factor that was significant despite the starting hypothesis that was being considered.

It is essential to contextualize the research in the Spanish territory with respect to the proposed government measures. Specifically, the removal of the face mask was widely accepted due to the amount of information released by the media that warned of the damage that wearing the mask could entail at the health level. In the first half of 2022, there was an avalanche of news of this type in the media that, coinciding with the dissemination of the online questionnaire, could bias the expected results.

If the results of the variables are unified as a whole: acceptance of confinement, belief in safety when wearing a mask, use of vaccines, the predictability of resilience increases. The measures proposed by the Spanish government cannot have been accepted by all citizens despite demanding responsible and civic action. It is necessary to be aware that political beliefs have been able to create rejection of the proposed measures, despite their effectiveness and relationship with resilience. Perhaps this study should consider being sympathetic to the political party that governs during the pandemic as a predictor of better acceptance of the government measures proposed to stop the virus.

The analysis of all the obtained information has as its main objective to be able to demonstrate the predictability of resilience through civic and responsible attitudes that lead us to the acceptance of government regulations in catastrophes or emergencies, such as pandemic, that we are going through. Thanks to the different results obtained in most age ranges and in any of sexes, a significant relationship between the studied variables is demonstrated.

This research involves a reflection that mobilizes actions aimed at promoting and creating preventive programs that develop protective psychological factors in adverse situation as a pandemic. Although the research was based on the predictive relationship between acceptance of measures to mitigate virus transmission and resilience, it is essential to emphasize that we were talking about attitudes; in particular, seeking a degree of agreement with the measures: use of masks, vaccines and confinement. For this reason, one of the main limitations would be those participants who, despite having an attitude of respect to the measures proposed to stop pandemic, have had the opposite behavior, and therefore present a discrepancy between their thoughts and behaviors.

This limitation forces to talk about attitudes towards the virus and not so much about observable and real behaviors; although we could predict an adequate behavior in the face of the measures, we cannot affirm it outright. This should be considered for future researches.

The results of this research could be in line with First & Houston [6], who published that the development of resilience directly affects good coping with potentially traumatic situations. This encompasses a close relationship with the study since a positive relationship was found between positive attitude to anti-covid measures (vaccines and confinement) and high resilience, and less relationship between wearing masks and high resilience.

Studies about workers’ behavior during this pandemic, like Bozdag & Ergün [23]. concluded that resilience improved their responsible behavior and mental health, so then they are in the same line of results of this study. The conclusion being that resilience is related to coping well with critical situations and preserving mental health. That hypothesis was similar to the one proposed in this research.

The results obtained also reinforce the conclusions of the research carried out by Kocjan and his team of researchers [28] who saw evidence at the relationship between personality, psychological functioning and high resilience during the COVID-19 pandemic. In particular, that study showed how resilience mediated the relationships among the Big Five (specifically, extraversion with subjective well-being, stress experienced and neuroticism predictor of less resilience).

The hypotheses proposed by Prentice et al. [20] and Sharif et al. [29] are also more specifically corroborated, despite the fact that their team of researchers focused their attention on more generic and complex aspects. Emotional intelligence, attitude, coping with the pandemic and personality traits; among which those compatible with those that describe a person with high resilience stood out. Emotional intelligence; is significantly related to all coping strategies in the face of catastrophic situations such as the pandemic.

It is necessary to consider some factors that have been able to bias this research, such as a misinterpretation of the items raised in the questionnaire, as well as problems in the personal or clinical history of the chosen participants that may lead to conclusions that are far from reality. This can be seen, for example, at the percentage of people with disabilities, older people or participants with several mental health problems who completed the questionnaire; and at that sample, the perception could be altered or could show cognitive deterioration not contemplated at the results.

This study had some limitations. Among them, it is important to highlight that the sample obtained was mainly formed by people who had access to Internet and management of technology, so that the majority of people could be considered young people.

This could imply that the older adult population had little representation in this study.

The lower participation of older people could be interpreted in relation to their difficulties in accessing an online form, such as the one used in this study. If considered, as some studies indicate, a great intergenerational digital gap exists in Spain.

Despite being a limited sample of participants, exclusively based on the Spanish adult population and with a young average age, the results are very enlightening and able to continue developing tools that improve people’s quality of life; especially in the most critical moments, because it is where they need to get the best of each human being.

With the present research a predictive relationship according to the measures proposed by the government on the use of masks, vaccination, confinement and high levels of resilience has been shown. These conclusions could contribute to make efforts to create awareness campaigns in the face of crises and emergencies. These campaigns in order to promote civic and responsible attitudes so as to face of adversity which that may interfere with an increase in their resilience and therefore also in their mental strengths necessary to preserve their mental health.

Although psychology has traditionally focused on the study of vulnerabilities, mental illnesses and factors that influence the detriment of mental health, we believe it is necessary to continue with the efforts of many psychology researchers in the prevention and development of potentialities that improve adaptability from the human being to the unpredictable future.

The suffering and psychological affectation that the presence of this virus has caused in our lives is undeniable. A lot of authors have contributed to analyze the increase in mental illnesses and health problems associated with this pandemic [30, 31]. Also, it is necessary once again to analyze the reasons why people who have suffered a lot have been able to come out stronger and much more resilient.

Therefore, working on the analysis of the factors that can predict resilience in a certain way is basic and marks our line of research for the coming years. Resilience encompasses basic elements of survival and eudemonic happiness, such as self-knowledge and self-esteem, empathy, autonomy, optimism, flexibility, tolerance of uncertainty. It is worth to continue efforts towards its investigation and prediction. Recents researches have studied the variables involved in such a process of personal growth are seen [31–35].

It is essential to discover that we are prepared to withstand crises, support human suffering, as well as drawing courage in the worst of battles. pandemic is not over and, therefore, there is still much to discover and relearn in these times of reflection.

## Electronic supplementary material

Below is the link to the electronic supplementary material.

Supplementary Material 1

Supplementary Material 2

## Data Availability

The present study used publicly available secondary de-identified data and this did not involve human subjects. Publisher’s note remained neutral with regard to jurisdictional claims in published maps and institutional affiliations. The data sets generated and/or analyzed during the present study are not publicly available due to confidentiality and privacy related issues but are available from the corresponding author upon reasonable request.
