# Supplementary material for: Experiential COVID-19 factors predicting resilience among Spanish adults
Source: BMC Psychol. 2023 Apr 17;11:118. doi: 10.1186/s40359-023-01131-4 (PMC10108776; doi:10.1186/s40359-023-01131-4)
Supplement: Supplementary file 2 — Supplementary Material 2 [file 40359_2023_1131_MOESM2_ESM.docx]

| **Correlations** | | | | | | | |
| --- | --- | --- | --- | --- | --- | --- | --- |
|  | | 4) vaccines | 8) Feel strong by the Pandemic | 18) Safe of masks | Resilience | sex = 1 (FILTER) | age |
| 4) vaccines | Correlation of Pearson | 1 | ,096^**^ | ,297^**^ | ,279^**^ | -,042 | ,000 |
|  | Sig. (bilateral) |  | ,002 | ,000 | ,000 | ,185 | ,993 |
|  | N | 1000 | 1000 | 1000 | 1000 | 998 | 999 |
| 8) Feel strong by the Pandemic | Correlation of Pearson | ,096^**^ | 1 | ,077^*^ | ,142^**^ | -,108^**^ | ,055 |
|  | Sig. (bilateral) | ,002 |  | ,015 | ,000 | ,001 | ,080 |
|  | N | 1000 | 1000 | 1000 | 1000 | 998 | 999 |
| 18) Safe of masks | Correlation of Pearson | ,297^**^ | ,077^*^ | 1 | ,277^**^ | ,121^**^ | ,145^**^ |
|  | Sig. (bilateral) | ,000 | ,015 |  | ,000 | ,000 | ,000 |
|  | N | 1000 | 1000 | 1000 | 1000 | 998 | 999 |
| Resilience | Correlation of Pearson | ,279^**^ | ,142^**^ | ,277^**^ | 1 | ,112^**^ | -,097^**^ |
|  | Sig. (bilateral) | ,000 | ,000 | ,000 |  | ,000 | ,002 |
|  | N | 1000 | 1000 | 1000 | 1000 | 998 | 999 |
| sex = 1 (FILTER) | Correlation of Pearson | -,042 | -,108^**^ | ,121^**^ | ,112^**^ | 1 | -,080^*^ |
|  | Sig. (bilateral) | ,185 | ,001 | ,000 | ,000 |  | ,012 |
|  | N | 998 | 998 | 998 | 998 | 998 | 997 |
| age | Correlation of Pearson | ,000 | ,055 | ,145^**^ | -,097^**^ | -,080^*^ | 1 |
|  | Sig. (bilateral) | ,993 | ,080 | ,000 | ,002 | ,012 |  |
|  | N | 999 | 999 | 999 | 999 | 997 | 999 |
| **.The correlation is relevant in level 0,01 (bilateral). | | | | | | | |
| *. The correlation is relevant in level 0,05 (bilateral). | | | | | | | |
